# Laser-Induced Axotomy of Human iPSC-Derived and Murine Primary Neurons Decreases Somatic Tau and AT8 Tau Phosphorylation: A Single-Cell Approach to Study Effects of Acute Axonal Damage

**DOI:** 10.1007/s10571-023-01359-z

**Published:** 2023-05-12

**Authors:** M. Bell-Simons, S. Buchholz, J. Klimek, H. Zempel

**Affiliations:** 1 Institute of Human Genetics, University Hospital Cologne, Kerpener Str. 34, 50931 Cologne, Germany; 2 Center for Molecular Medicine Cologne (CMMC), Robert-Koch-Str. 21, 50931 Cologne, Germany

**Keywords:** Axotomy, Tau sorting, Tau pathology, Tau phosphorylation, Alzheimer’s disease, Tauopathies

## Abstract

The microtubule-associated protein Tau is highly enriched in axons of brain neurons where it regulates axonal outgrowth, plasticity, and transport. Efficient axonal Tau sorting is critical since somatodendritic Tau missorting is a major hallmark of Alzheimer’s disease and other tauopathies. However, the molecular mechanisms of axonal Tau sorting are still not fully understood. In this study, we aimed to unravel to which extent anterograde protein transport contributes to axonal Tau sorting. We developed a laser-based axotomy approach with single-cell resolution and combined it with spinning disk confocal microscopy enabling multi live-cell monitoring. We cultivated human iPSC-derived cortical neurons and mouse primary forebrain neurons in specialized chambers allowing reliable post-fixation identification and Tau analysis. Using this approach, we achieved high post-axotomy survival rates and observed axonal regrowth in a subset of neurons. When we assessed somatic missorting and phosphorylation levels of endogenous human or murine Tau at different time points after axotomy, we surprisingly did not observe somatic Tau accumulation or hyperphosphorylation, regardless of their regrowing activity, consistent for both models. These results indicate that impairment of anterograde transit of Tau protein and acute axonal damage may not play a role for the development of somatic Tau pathology. In sum, we developed a laser-based axotomy model suitable for studying the impact of different Tau sorting mechanisms in a highly controllable and reproducible setting, and we provide evidence that acute axon loss does not induce somatic Tau accumulation and AT8 Tau phosphorylation.

**Graphical Abstract:**

UV laser-induced axotomy of human iPSC-derived and mouse primary neurons results in decreased somatic levels of endogenous Tau and AT8 Tau phosphorylation. 
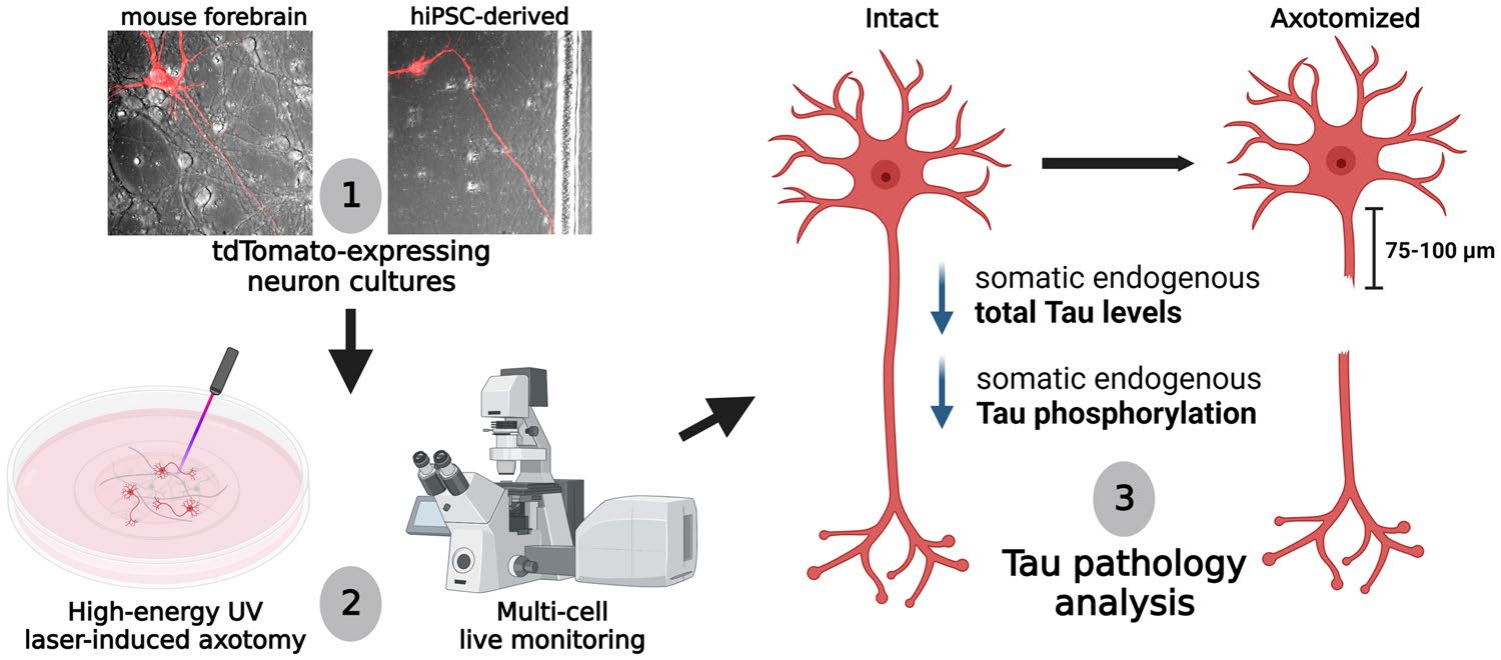

**Supplementary Information:**

The online version contains supplementary material available at 10.1007/s10571-023-01359-z.

## Introduction

Under healthy conditions, the microtubule-associated Tau protein is targeted to the axonal compartment of mature brain neurons where it regulates the assembly and dynamics of microtubule filaments. Thereby, Tau is involved in axonal outgrowth, plasticity, cargo transport, and other essential cellular functions. Pathological alterations of Tau constitute a major hallmark of many neurodegenerative diseases (Arendt et al. 2016). In these so-called tauopathies, hyperphosphorylation and somatodendritic missorting of Tau leads to i) Tau-mediated loss of dendritic MT filaments and spines, and ii) impaired axonal MT-mediated transport (Roy et al. 2005). One possible strategy to counteract or ameliorate pathological missorting in tauopathies may be boosting the physiological processes of axonal targeting.

However, the processes underlying physiological Tau sorting are still not fully understood (Zempel and Mandelkow 2019). Several studies suggest that active anterograde trafficking of Tau constitutes a major mechanism of Tau sorting (Konzack et al. 2007; Zempel et al. 2013; Scholz and Mandelkow 2014). Selective retrograde retention of axonally targeted Tau seems to further elevate axonal Tau levels. Studies in primary rodent neurons found a Tau diffusion barrier within the axon initial segment (AIS) (Li et al. 2011; Zempel et al. 2017a) and impaired mobility of axonal Tau due to microtubule binding (Kanai and Hirokawa 1995; Zempel et al. 2010; Zhu et al. 2010). Besides that, increased somatodendritic degradation of Tau is thought to support Tau polarized distribution (Balaji et al. 2018). Apart from these protein-centric explanations, there is also evidence for preferential axonal localization and/or translation of the Tau mRNA (Litman et al. 1993; Aronov et al. 2001; Morita and Sobuě, 2009). Also, despite the high dynamics of Tau-microtubule interactions, the high density of interaction sites in the axon of polarized neurons combined with mechanisms that promote axonal retention, e.g., by binding to annexins (Arendt et al. 2016), could generate Tau enrichment in the axon without the need of a net anterograde transport machinery. We have previously detailed both possible mechanisms and consequences for missorting (Zempel and Mandelkow 2014) and sorting (Zempel and Mandelkow 2019), but more detailed studies are necessary to decipher the relevance of these mechanisms on Tau sorting.

Interestingly, our system may also serve as a model for a subset of tauopathies that occur due to acute or chronic mechanical impact, such as traumatic brain injury (TBI) or chronic traumatic encephalopathy (CTE) (Gennarelli et al. 1982; Gentleman et al. 1995; Geddes et al. 2000; Tran et al. 2011). In these diseases, the physical harm leads to axonal injury that in turn results in Tau hyperphosphorylation, missorting, and formation of neurofibrillary tangles (Dale et al. 1991; Tokuda et al. 1991; Ikonomovic et al. 2004; Uryu et al. 2007; Goldstein et al. 2012). As most available models use diffuse triggers for mimicking mechanical impact, our single-cell based approach bears great potential to address molecular changes and malfunctions after the axon injury.

Here, we describe an axotomy model suitable to study, e.g., the effect of physical axonal disruption (resulting also in complete block of anterograde Tau trafficking) on Tau and consequences for Tau distribution and other somatodendritic cellular effects, like dendrite outgrowth, on a single-cell level and in a pulse-chase fashion. The experimental setup consists of a high-energy UV ablation laser combined with a high-speed spinning disc microscope enabling multi live-cell monitoring under environmentally controlled conditions. Our approach has three major benefits for future Tau sorting/behavior studies: (I) We successfully applied it to rodent primary neurons, a widely used model for Tau sorting studies and human iPSC-derived cortical neurons, a newly established neuronal model for Tau studies. (II) We used specialized cultivation chambers that allowed for post-fixation identification and analysis of endogenous Tau accumulation and AT8 phosphorylation without the need of exogenous Tau overexpression. III) The precise laser-induced axon severing enables the observation of (somatodendritic) Tau behavior without soma-to-axon transit of Tau protein or mRNA molecules on single-cell resolution without notable neuronal loss.

Using our setup, we determined potential changes in somatic Tau levels and its phosphorylation state after axotomy and at different time points of fixation. Our data show that acute axon loss does not induce somatic accumulation or hyperphosphorylation of Tau in both neuronal model systems independent of axon regrowth. This hints towards little importance of axonal damage to the development of somatic Tau accumulation and AT8 phosphorylation.

## Methods

### Molecular Biology

For experiments with endogenous Tau, an expression vector harboring tdTomato-encoding cDNA was used as volume marker control. For plasmid amplification, plasmids were transformed into chemocompetent bacteria (*E. coli* Top10™, ThermoFisher Scientific) that were generated like previously described (Sambrook and Russell 2006). Bacteria were spread onto pre-warmed agar plates with antibiotics (16–18 h, 37 °C), colonies were picked and grown in nutrient medium with antibiotics. For plasmid isolation and purification from bacterial suspensions, the PureYield™ MidiPrep Kit (ProMega) and NucleoBond Xtra™ Midi/Maxi Kit (Macherey & Nagel) were used according to the manufacturer’s protocols. Plasmid concentration and purity were checked spectrophotometrically (NanoDrop™ 100, ThermoFisher Scientific) and isolates were long-term stored at − 20 °C.

### Cultivation of iPSC-Derived Neurons

#### Maintenance

The human iPSC cell line WTC11 carrying a doxycycline-inducible Ngn2 transgene (Miyaoka et al. 2014; Wang et al. 2017) were cultivated as previously described (Bell et al. 2021; Buchholz et al. 2022). Briefly, iPSCs were cultured on GelTrex™-coated plates (1X, ThermoFisher Scientific) in StemMACS™ iPS-Brew XF (Miltenyi). When reaching confluence, the cultures were passaged with Versene™ passaging solution (ThermoFisher Scientific) and seeded in thiazovivine-supplemented (Axon Medchem) iPS-Brew for 1 day. Cells were grown at 37 °C and 5% CO_2_ in a humidified incubator.

#### Differentiation

Differentiation into cortical neuronal cultures was performed as previously described (Bachmann et al. 2021; Buchholz et al. 2022). Briefly, cells were seeded in high density onto GelTrex-coated plates using pre-differentiation medium supplemented with thiazovivine (day-3). The pre-differentiation medium was refreshed daily for 2 days with thiazovivine-free pre-differentiation medium. On day 0, cells were seeded on grid-containing cultivation chambers (#81168, ibidi) coated with 50 µg/ml Poly-d-Lysine (PDL, Sigma Aldrich) and 20 µg/ml Cultrex™ 3D-laminin (Sigma Aldrich) in maturation medium supplemented with 1:100 GelTrex. Once per week, half of the medium was replaced by fresh GelTrex-free maturation medium.

#### Transfection

Human iPSC-derived cortical neurons were transfected with a tdTomato-expressing plasmid at day 11 (d11). For transfection, 0.5 µg of plasmid DNA and 1 µl Lipofectamine™ Stem (#STEM0001, Invitrogen) were mixed for one well of a 24-well plate, incubated for 10 min in pre-warmed Opti-MEM™ (Invitrogen) and added dropwise to the cultures. Medium was changed to previously collected medium 24 h after transfection.

### Cultivation of Primary Mouse Neurons

#### Isolation

Pregnant female FVB wild type mice were anesthetized and sacrificed at day 13.5 of pregnancy by authorized personnel (§4 approval numbers 4.19.014 & 4.19.016) with respect to the official regularities and guidelines of the governmental authority, the Landesumweltamt (LANUV) of North Rhine-Westphalia, Germany. Preparation of E13.5 embryos, and isolation and cultivation of primary neurons were performed like previously described (Zempel and Mandelkow 2017). In brief, embryos were decapitated, embryonic scalp, skull and meninges were removed, the cerebral hemispheres were isolated, washed, dissociated for 5–7 min with 0.05% Trypsin/EDTA, and homogenized in HBSS (w/o Ca^2+^/Mg^2+^, #14185052, ThermoFisher Scientific). Dissociated primary cells were counted with the automated cell counter TC20™, (Bio-Rad) using trypan blue (#1450022, Bio-Rad) and seeded with 5 to 7.5 × 10^4^ cells/cm^2^ in neuronal plating medium (NPM), i.e. Neurobasal™ medium (#21103, ThermoFisher Scientific) supplemented with 1% FBS, 1X Anti/Anti (ThermoFisher Scientific), 1X GlutaMAX™ (ThermoFisher Scientific) and 1X NS-21 (#P07-20001, PAN Biotech), onto glass coverslips, which were previously coated with 20 µg/ml PDL for at least 3 h at 37 °C.

#### Maintenance

Four days after seeding, the medium was doubled by adding neuronal maintenance medium (NMM), i.e. NPM without FBS. Cytosine arabinoside (Ara-C, #C1768, Sigma Aldrich) was added to a final concentration of 0.5–1.0 µg/ml to impair survival of glial, endothelial and other proliferating cells.

#### Transfection

Mouse primary forebrain neurons were transfected with a tdTomato-expressing plasmid at day 6 in vitro (div6). Transfection was performed as previously described (Zempel et al. 2017b). In brief, 0.25 µg of plasmid DNA and 0.375 µl Lipofectamine™ 2000 (#743517, Invitrogen) were mixed for one well of a 24-well plate, incubated for 30 min and added dropwise to the cultures. Medium was changed to previously collected medium 60 min after transfection.

#### Axotomy

Three days after transfection, i.e. at d14 (iPSC-derived neurons) or div9 (mouse primary neurons), the cultivation chambers were transferred to the incubation chamber (Solent Scientific) with controlled temperature and atmosphere conditions, at least 30 min prior to axotomy to equilibrate. TdTomato-expressing neurons were depicted and either used as control neurons or axotomized. For axotomy, a high-energy UV laser (405 nm) point illumination system (UGA-40, RappOptoElectronic) was used. The line-shaped cutting site was applied 75–100 µm distal of the somatic compartment. The laser settings were modulated for each neuron. In general, a run of ten pulses of 100 ms duration was repeated up to four times, the laser power was increased and/ a second linear ROI was drawn between the runs in case of incomplete severance. The laser power did not exceed 30% of maximum during all experiments.

#### Live-Cell Monitoring

The long-term live-cell monitoring was performed with an inverse EclipseTi™ fluorescence microscope (Nikon) linked to the UltraView™ Vox spinning disc device (PerkinElmer) and modular excitation lasers (PerkinElmer), using the Volocity™ imaging software (Quorum Technologies). The coordinates of each depicted neurons were stored for multi live-cell monitoring prior to axotomy, and all neurons were monitored from before (*t* = 0) and after the axotomy for either 30 min, 60 min (interval between images: 10 min), 3 h (interval: 20 min) or 12 h (interval: 1 h). The high scanning velocity of the spinning disc technology allowed to record z-stacks of 25–30 µm thickness from up to 20 cells at each time point to compensate for potential focus drift.

#### Fixation & Immunofluorescence

Fixation and immunofluorescence of cell cultures were done as described previously (Zempel et al. 2017b) with slight modifications. In brief, cells were fixed by adding 7.4% formaldehyde (FA) solution to the culture chamber while it was still placed in the microscope incubator. The FA solution was added to the chamber dropwise with constant monitoring of the culture on the computer screen until a final concentration of 3.7% FA was reached. Note: Removing of the culturing chamber and adding the fixation solution outside the incubation chamber failed, as vast portions of the cultured neurons appeared displaced. After 30 min, the medium-FA mix was removed manually, and cells were washed once with PBS and covered with 60% glycerol solution. The cultivation chamber was removed from the microscope and stored at − 20 °C. For immunostaining, the glycerol was removed, and the neurons were permeabilized and blocked with 5% bovine serum albumin (BSA, Sigma-Aldrich) and 0.1% Triton X-100 (AppliChem) in PBS for 5 min, incubated with the primary antibodies (diluted in PBS) either for 2–3 h at 20–22 °C or preferentially at 4 °C for 16–18 h. After thorough washing steps, the cultures were incubated with the corresponding secondary antibody (diluted in PBS), coupled to AlexaFluor™ fluorophores for 1–2 h at 20–22 °C. The following primary antibodies were used: rabbit anti-TAU (K9JA) (1:1000, #A0024, DAKO), mouse anti-phospho Tau (AT8) (1:1000, #MN1020, ThermoFisher Scientific). The specificity of all used primary antibodies was not tested within our study, but the validity of these well-established anti-Tau antibodies was demonstrated multiple times before (Goedert et al. 1995; Wang et al. 2007). Nuclei were stained with NucBlue™ (1 drop/ml, Hoechst 33342, ThermoFisher Scientific) for 20–30 min, and cultures were mounted within the chamber using aqueous PolyMount™ mounting medium (#18606, Polysciences), dried for 24 h at RT and long-term stored at 4 °C in the dark.

#### Post-staining Microscopy

The immunostained and mounted cultivation chambers were imaged with the confocal spinning disc microscope setup that was used for live-cell monitoring (see section live-cell monitoring). With few exceptions (< 5% of cells), all previously monitored axotomized and control neurons could be identified using the grid coordinates. Neurons were imaged using four excitation laser wavelengths: 405 nm (NucBlue), 488 nm (total Tau detected with AF™488), 561 nm (tdTomato), and 640 (phospho-Tau detected with AF™647). Laser power and exposure times were adjusted to ensure optimal visualization of all neurons considering the immunostaining signals. Once determined, the settings were kept constant for all neurons of one chamber. The somata were imaged using z-stacks with a step size of 1–1.5 µm.

#### Data Analysis

In general, all image files obtained from live-cell monitoring or post-staining microscopy were exported and transferred in the file format MVD2 and further processed using Fiji/ImageJ (Version 1.53i, NIH). For further analyses, the z-stacks of each recording were converted to maximum intensity projections and analyzed without randomization.

#### Survival & Regrowth Rate

For each axotomized neuron, the viability status was assessed to determine the overall survival rate of human iPSC-derived and mouse primary neurons at different time points after axotomy. Neurons were categorized as ‘viable’ when no morphological signs for cell degradation like detachment, axonal swellings, or cytosolic release were visible after the live-cell monitoring. Neurons that showed one or several of these alterations, they were considered as ‘dying’. Neurons that showed modest detachment without other signs of degradation were considered as ‘dysmorph’.

For each axotomized neuron categorized as ‘viable’, the regrowth activity was assessed. Neurons were considered ‘regrowing’ when a growth cone and axonal elongation were visible at the axotomized axon during live-cell monitoring. All other neurons were considered ‘no regrowing’. For a minor fraction, another than the cut process showed visible regrowing upon axotomy (‘regrowing of other process’).

#### Somatic Pan-Tau & Phospho-Tau Levels

The absolute somatic levels of total Tau and hyperphosphorylated Tau were measured using maximum intensity projections for each channel. For each neuron, the region of interest (ROI) was manually determined as a section of the soma where no other processes or somata were visible. A second ROI was measured close to the soma, and the intensity signal was subtracted from the somatic ROI. To evaluate the relative phosphorylation state of somatic Tau, the ratio of phospho-Tau to total Tau signals were calculated per neuron. Besides axotomized and tdTomato-expressing control neurons, also a set of non-transfected neurons was depicted for quantification of Tau and phospho-Tau levels for each time point to exclude Tau pathology and/or bleed-through effects of tdTomato light emission to adjacent channels.

### Statistical Analysis

All statistical calculations were performed using PRISM™ analysis software (V9.3.1, GraphPad Inc.). The normal distribution was tested by applying a Shapiro–Wilk normality test. For comparisons between two (control vs. axotomized neurons) or three normally distributed groups (tdTom-negative, tdTom-positive control, tdTom-positive axotomized) of samples at different time points (0.5 h, 1 h, 3 h), an ordinary two-way ANOVA with Sidak’s multiple comparison test was performed for the determination of significant differences. Exact p-values with four decimals are given for every performed analysis in the corresponding graph. Differences were considered as statistically significant if the p-value was p < 0.05. 

The sample size per experimental was determined with regard to the technical limitations of the setup, i.e. not too many neurons were used per chamber to ensure equal or almost equal monitoring times. For each time point except *t* = 30 min, in which two cultures were used per model system, one culture was investigated per model system.

## Results

### Establishment of a UV Laser-Based Axotomy Model in Human and Mouse Neuronal Cultures

**Fig. 1:**
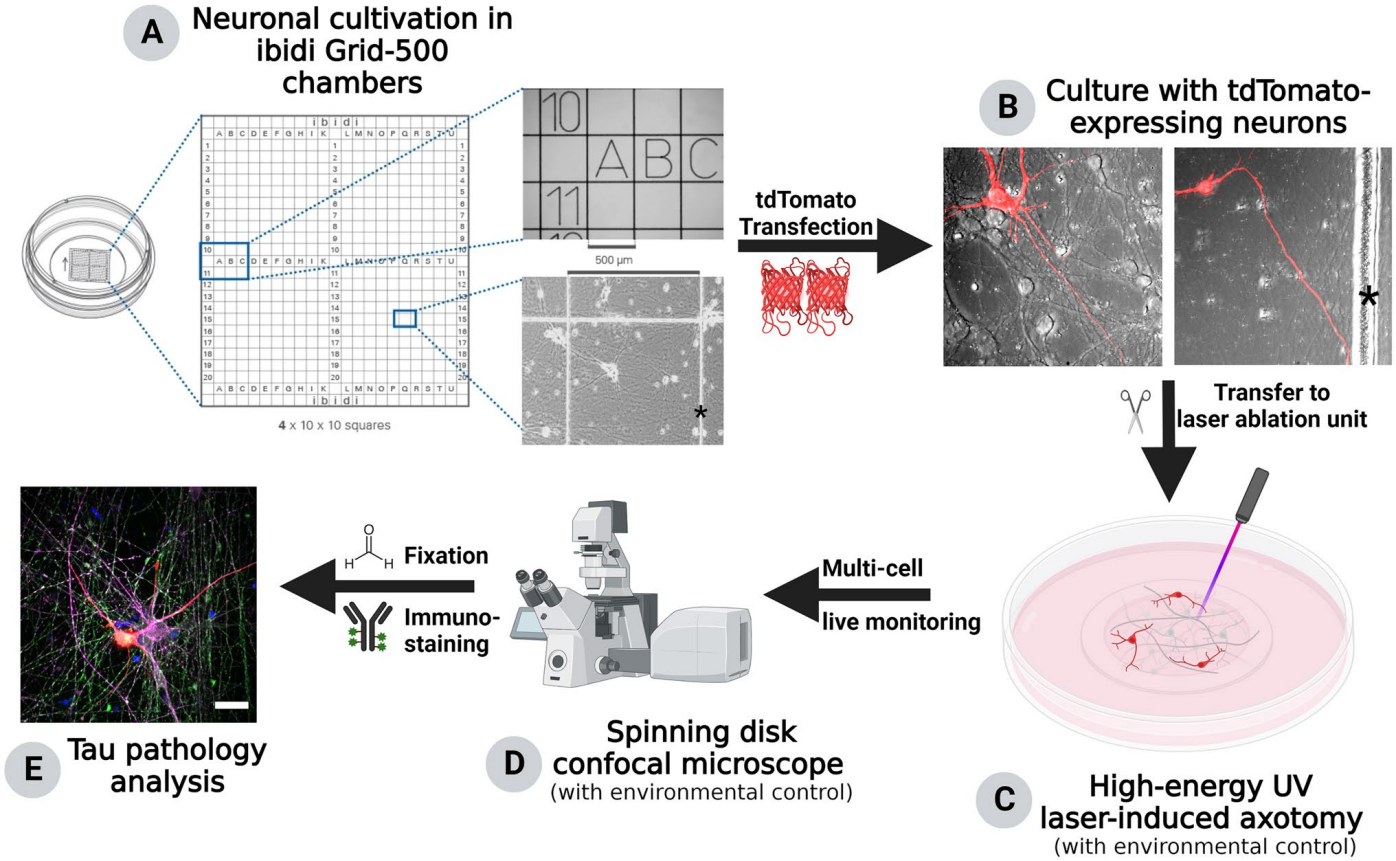
Experimental workflow of laser-based axotomy. Step **a**: Both human iPSC-derived and mouse primary neuron cultures are seeded and differentiated/grown in specialized imaging chambers (left pane) that contain a glass bottom with coordinate systems of 500 µm square size (middle & upper right). Human iPSC-derived cortical neurons are differentiated for 2 weeks (lower right), mouse primary neurons are grown until day 9 in vitro. The illustration was adapted from the manufacturer’s website (ibidi) and slightly modified. Step **b**: Neurons are transfected with tdTomato-expressing plasmid 3 days prior to axotomy resulting in mosaic transfection for better identification of neuronal processes. **a** and **b**: The asterisk marks a grid line visible in this image. Step **c**: The cultivation chambers are transferred to a spinning disk confocal microscope connected with a high energy UV laser ablation unit. Using a pulsed high-energy UV laser, the proximal axon of pre-selected tdTomato-positive neurons are severed (see Fig. 2). Step **d**: Multiple axotomized and control neurons are monitored before fixation for different time periods up to 12 h. Step c and d are performed under environmental control (5% CO_2_, 37 °C). Step **e**: After monitoring, cells are fixed within the incubation chamber and immunostained with multiple antibodies for subsequent analysis. Scale bar: 30 µm. Illustration was generated with Biorender

Although efficient axonal targeting of Tau is critical for normal neuronal functionality, the mechanisms underlying Tau sorting are still under debate. In our study, we aimed to develop a novel experimental strategy that allows for precise axotomy induction on a single-cell level combined with multi live-cell monitoring and subsequent analysis of Tau pathology, in our case determined via Tau AT8 phosphorylation and Tau missorting reminiscent of human disease conditions. We used two state-of-the-art neuronal cell culture models to evaluate our approach: (I) Human iPSC-derived cortical neurons differentiated from a Ngn2 transgene-harboring derivative of the iPSC line WTC11 (Miyaoka et al. 2014; Wang et al. 2017), and (II) primary forebrain neurons from embryonic day 13.5 FVB/N mice. The cultures were grown for up to 2 weeks (iPSC-neurons) or 9 days (primary neurons) in cultivation chambers with a built-in coordinate system that allows later (post-fixation) re-identification and analysis of axotomized neurons (Fig. 1a). Three days prior to axotomy induction, the neurons were transfected with a tdTomato-expressing plasmid. The low transfection efficiency of the liposome-based transfection method used here (see methods for details) facilitated the identification of individual neurons (Fig. 1b). For axotomy, the cultivation chambers were transferred to an incubation chamber connected to a high-energy UV laser ablation unit (Fig. 1c). TdTomato-expressing neurons were selected based on the described quality criteria (see methods for details) and either axotomized or kept untreated as control cells. The laser settings (i.e. laser intensity, repetitions, cutting ROI) were adjusted for each neuron. After axotomy, all axotomized and control neurons were monitored in parallel utilizing a spinning disk confocal microscope setup (Nikon, PerkinElmer) and monitored for different time periods up to 12 h (Fig. 1d). After fixation, neurons were immunostained and re-identified using their grid position for Tau missorting/AT8 phosphorylation analysis (Fig. 1e).

**Fig. 2:**
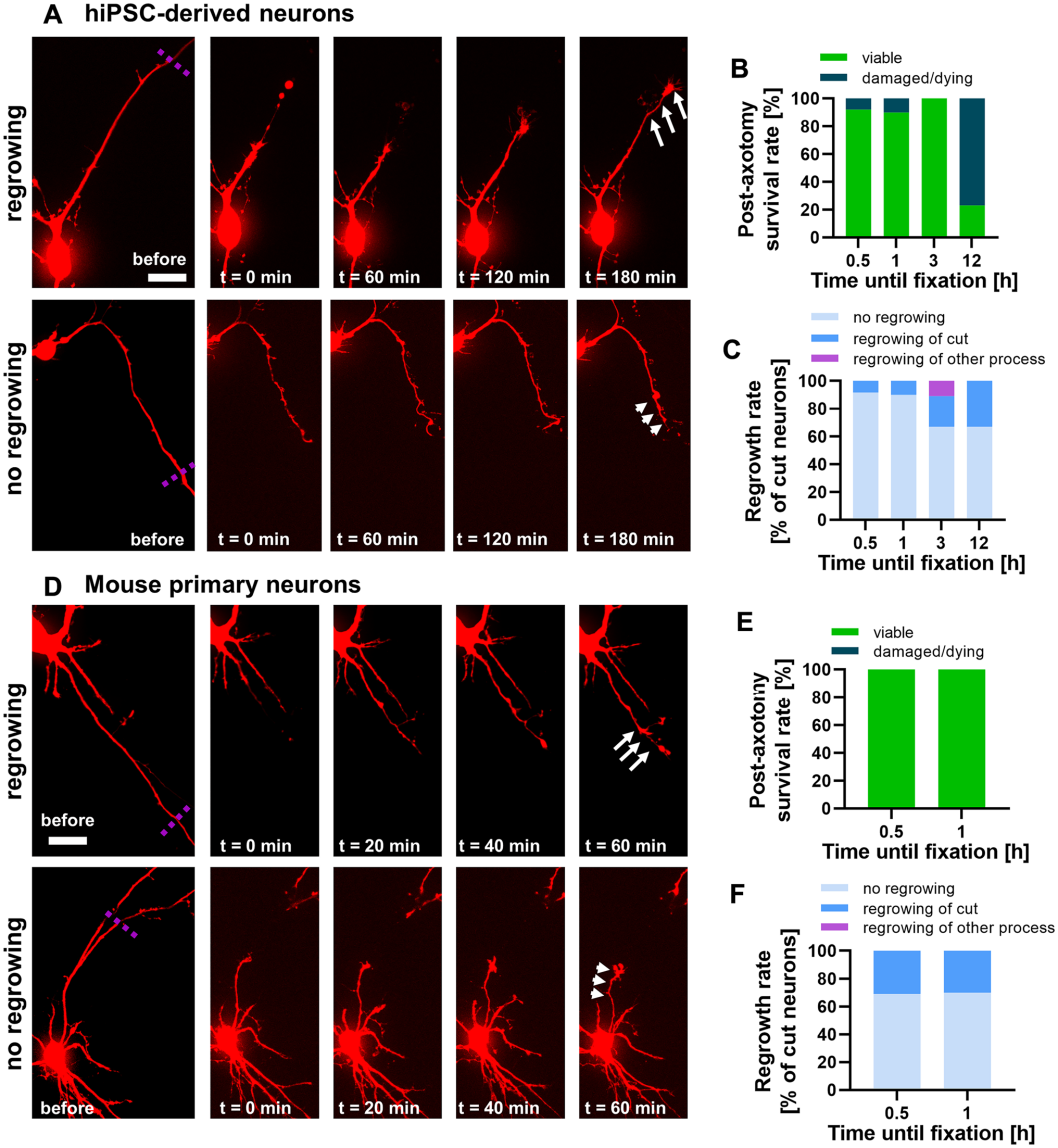
Survival rates and axonal regrowth of human iPSC-derived and mouse primary neurons after axotomy. Neurons were cultivated in imaging chambers either for 2 weeks (iPSC-neurons) or 9 days (primary neurons) and transfected with a tdTomato-expressing plasmid 3 days prior to the axotomy. Only tdTomato-positive neurons were selected for axotomy experiments. **a** and **d**: Two human iPSC-derived neurons (**a**) and two mouse primary neurons (**d**) before the UV laser induction (left panel, dotted violet lines indicate location of axotomy) and during post-axotomy monitoring for 3 h (right panels). In both cases, the axons (arrows) of the upper neurons regrow visibly while the axons (arrowheads) of the lower neurons do not show regrowing. Scale bars (refer to all panels in **b** and **d**): 20 µm. **b** and **e**: Survival rates of axotomized iPSC-derived (**b**) and primary neurons (**e**) for different times until fixation. **c** and **f**: Regrowth rates of axotomized and viable iPSC-derived (**c**) and primary neurons (**f**) for different times until fixation

In both neuronal models previously shown to exhibit efficient axonal targeting of endogenous Tau by us and others (Zempel et al. 2017a; Iwata et al. 2019; Bell et al. 2021), tdTomato-expressing neurons were selected for axotomy experiments. The axon was severed proximally but in sufficient distance to avoid lesions in the AIS region, i.e. 75 to 100 µm distance to the soma (Fig. 2a, d). Neurons were then monitored for different time periods ranging from 30 min up to 12 h for iPSC-derived neurons, and from 30 min to 1 h for mouse primary neurons, to check for viability status (Fig. 2a, d). To this end, neurite retraction, axonal swelling, and somatic detaching was assessed in axotomized compared to control neurons after the monitoring. For all neurons considered ‘viable’, no axonal swellings, somatic detaching or neurite retraction (for quantification: see Fig. S1) was observed. The large majority of iPSC-derived neurons (~ 90–100%) appeared ‘viable’ except for the 12 h post-axotomy monitoring, for which the majority of cells showed these signs and were considered ‘damaged/dying’ (Fig. 2b). Due to the low survival rate, the neurons of the 12 h post-axotomy monitoring were excluded. All axotomized primary neurons were judged as ‘viable’ during the monitoring (Fig. 2e). Next, we checked whether the viable neurons showed axonal regrowth post-axotomy. In the human iPSC-derived neurons, the regrowth rate increased with monitoring time reaching up to 40% after 3 or 12 h, but almost no regrowth (< 10%) was visible within the first hour (Fig. 2c). In rare cases (< 10%), a new neurite regrew upon axotomy (Fig. 2c). Primary mouse neurons showed considerably more axonal outgrowth after 30 min and 1 h, i.e. approximately 30% of neurons at each time point (Fig. 2f).

All in all, the high survival and re-identification rates in the first hours and the considerable axonal regrowth in both neuronal systems highlight our approach as a suitable methodology for investigating the effects of axotomy in both neuronal models on a single-cell level.

### Axotomized Neurons Do Not Show Increased Somatic Tau Accumulation and Phosphorylation Independent of Axonal Regrowth

Next, we asked whether axotomy results in impairment of axonal Tau trafficking. When lacking the axon as the target compartment, the anterograde transport should be compromised and lead to somatic accumulation of Tau protein assuming that Tau protein synthesis mainly occurs in the soma (Konzack et al. 2007; Zempel et al. 2013; Scholz and Mandelkow 2014; Zempel and Mandelkow 2019).Therefore, we assessed the somatic Tau levels and its phosphorylation state.

**Fig. 3:**
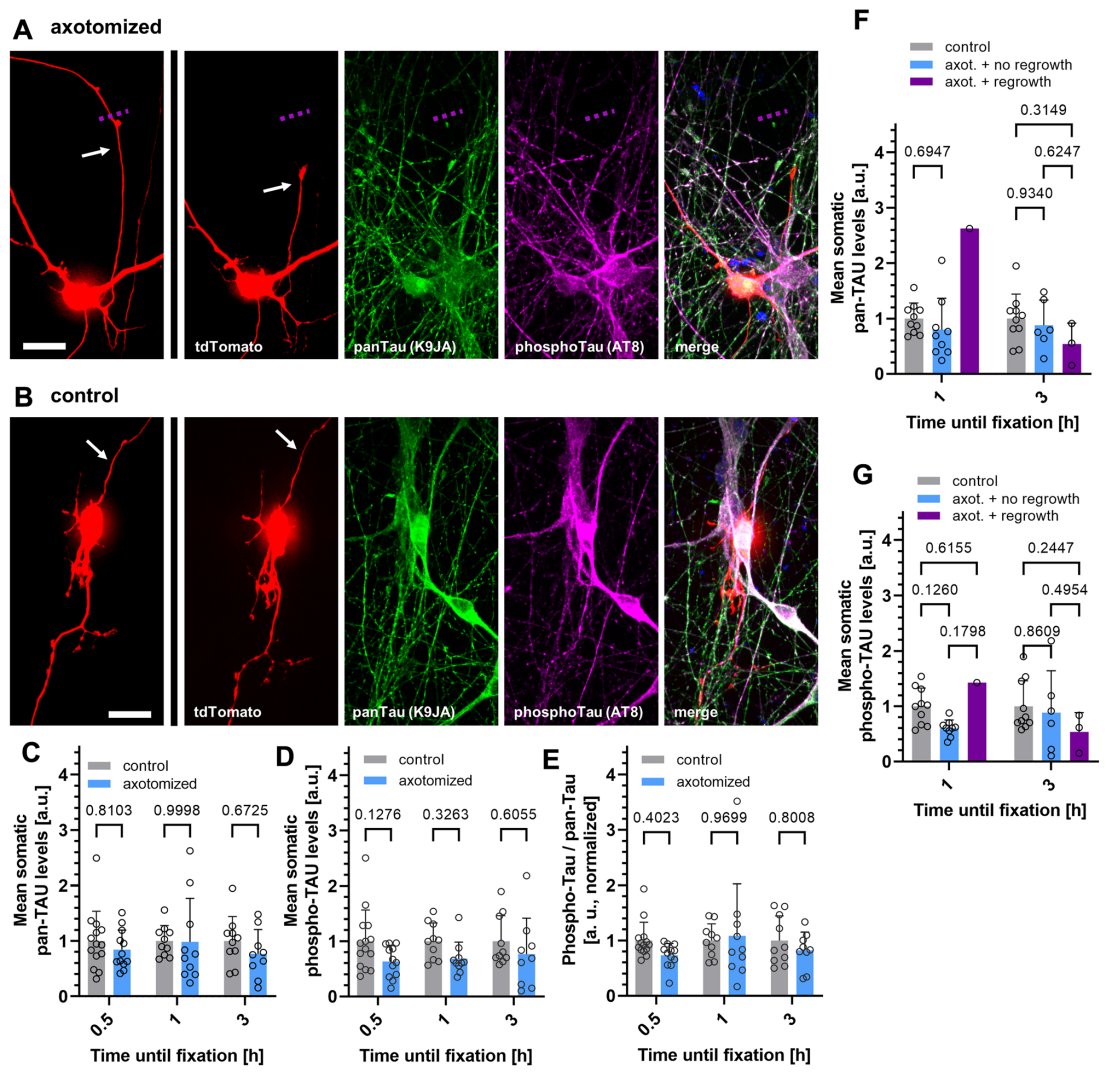
Somatic total Tau and phospho-Tau levels are not elevated after induced axon lesion in human iPSC-derived neurons independent of regrowth activity. Human iPSC-derived neurons were fixed after axotomy and subsequent monitoring at different time points (see methods for details), and immunostained with a polyclonal anti-panTau antibody (K9JA) and a monoclonal antibody recognizing Tau phosphorylation (AT8 epitope). **a** and **b**: Representative images of two iPSC-derived neurons that were grown, fixed, and immunostained in the same chamber, either after axotomy (**a**) or without axotomy (**b**) after 3 h monitoring. The left panels show the immunostained neurons before axotomy. The dotted violet lines indicate the location of axotomy. Scale bars (refer to all panels in **b** and **d**): 20 µm. **c** and **d**: Quantification of the total Tau (**c**) and phospho-Tau (**d**) signal intensity in the somata of axotomized neurons, normalized to the signals of non-axotomized neurons. **e**: Ratio of phospho-Tau and total Tau levels in the somata of axotomized neurons, normalized to the ratio of non-axotomized neurons. **f** and **g**: Total Tau (**f**) and phospho-Tau levels (**g**) in axotomized neurons depending on the regrowth after axotomy. Grey dots represent individual neurons, colored bars indicate the arithmetic mean of all neurons, error bars represent the standard deviation (SD). An ordinary two-way ANOVA with Sidak’s multiple comparison test was performed for the determination of significant differences. Exact adjusted p-values are given for all comparisons. For detailed test statistics: see Supplemental Material 1

**Fig. 4:**
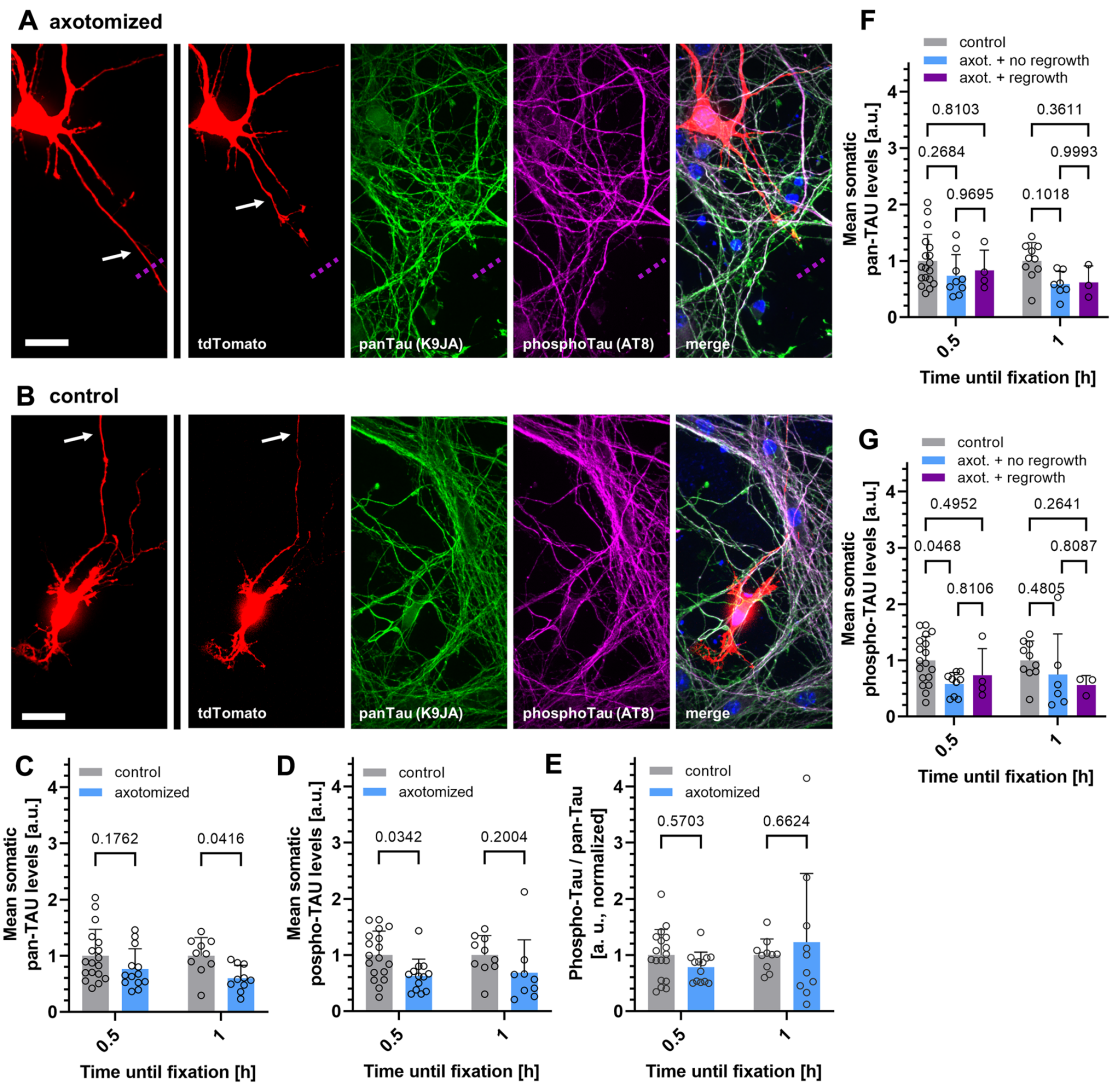
Somatic total Tau and phospho-Tau levels are not elevated after induced axon lesion in mouse primary forebrain neurons. Mouse primary forebrain neurons were fixed after axotomy and subsequent monitoring at different time points (see methods for details), and immunostained with a polyclonal anti-panTau antibody (K9JA) and a monoclonal antibody recognizing Tau phosphorylation (AT8 epitope). **a** & **b**: Representative images of two mouse primary neurons that were grown, fixed, and immunostained in the same chamber, either after axotomy (**a**) or without axotomy (**b**) after 3 h monitoring. The left panels show the immunostained neurons before axotomy. The dotted violet lines indicate the location of axotomy. Scale bars (refer to all panels in b&d): 20 µm. **c** and **d**: Quantification of the total Tau (**c**) and phospho-Tau (**d**) signal intensity in the somata of axotomized neurons, normalized to the signals of non-axotomized neurons. **e**: Ratio of phospho-Tau and total Tau levels in the somata of axotomized neurons, normalized to the ratio of non-axotomized neurons. **f** and **g**: Total Tau (**f**) and phospho-Tau levels (**g**) in axotomized neurons depending on the regrowth after axotomy. Grey dots represent individual neurons, colored bars indicate the arithmetic mean of all neurons, error bars represent the standard deviation (SD). An ordinary two-way ANOVA with Sidak’s multiple comparison test was performed for the determination of significant differences. Exact adjusted *p*-values are given for all comparisons. For detailed test statistics: see Supplemental Material 1

To this end, axotomized cultures were fixed 0.5, 1, 3 or 12 h after live-cell monitoring, and total Tau levels or phosphorylation at the AT8 motif, which is often hyperphosphorylated in tauopathies (Arendt et al. 2016), was assessed with immunostaining. Axotomized neurons were re-identified after staining via their grid position. In iPSC-derived, somatic levels of Tau and hyperphosphorylated Tau of axotomized neurons (Fig. 3a) were compared to those of tdTomato-positive but non-axotomized neurons as control (Fig. 3b). Quantification of Tau levels and AT8 phosphorylation in tdTomato-negative neurons was additionally performed for both murine and human neurons, for all time points, but showed overall no significant effect of transfection and fluorescent protein expression on Tau phenotypes. (Fig. S1). Comparing tdTomato-positive axotomized and control neurons, we observed no significant changes in the somatic levels of total Tau (Fig. 3c) or phosphorylated Tau (Fig. 3d) for any monitored time in iPSC-derived neurons, despite a trend towards decreased Tau phosphorylation visible at all time points. The ratio of phospho-Tau and total Tau was not significantly different indicating a similar phosphorylation state of somatic Tau among all groups (Fig. 3e). For primary mouse forebrain neurons, the analysis of somatic Tau levels and phosphorylation showed similar results than in iPSC-derived neurons, i.e. no increase in somatic Tau or AT8 phosphorylation (Fig. 4a–e). In contrast, the levels of total Tau (0.5 h) and Tau phosphorylation (1 h) were even significantly decreased for one time point each upon axotomy (Fig. 4c, d). The ratio of phospho-Tau to total Tau was not different to control neurons. Interestingly, total Tau levels and Tau phosphorylation did not correlate with the regrowth behavior of axotomized iPSC-derived or primary neurons (Fig. 3f, g, 4f, g).

In sum, we could observe a trend (iPSC-derived neurons, mouse primary neurons) or statistically significant differences (mouse primary neurons) towards reduced levels of total and AT8 phosphorylated Tau after axotomy, independent of the time point. Unexpectedly, we did not see any increase in somatic Tau levels or phosphorylation. These results strongly hint towards the absence of immediate effects on Tau pathology, here investigated via AT8 phosphorylation as the most accepted marker of human disease, and Tau missorting as an early disease marker) within the first hours after axotomy. Taken together, our data suggest absence of Tau pathology after acute axotomy.

## Discussion

Mislocalization of Tau into the somatodendritic compartment (‘Tau missorting’) is a key event of Tau pathology development, which is usually defined by the presence of AT8 phosphorylation, Tau missorting or ectopic presence, and Tau aggregation in many neurodegenerative diseases summarized under the term tauopathies. Despite the importance of subcellular Tau distribution in physiology (Zempel and Mandelkow 2019), and the failure thereof associated with human disease (Zempel and Mandelkow 2014), the mechanisms regulating efficient axonal Tau sorting in healthy neurons are still not well understood. Previous studies claimed active anterograde transport as one driving force of axonal Tau enrichment (Konzack et al. 2007; Zempel et al. 2013, 2017a; Scholz and Mandelkow 2014).

We developed an experimental approach that combines laser-based single-cell axotomy with multi live-cell monitoring and post-fixation analysis to study endogenous Tau and distribution in human iPSC-derived and mouse primary neurons. Our setup allows to evaluate intracellular Tau localization when anterograde transport is physically compromised in the most severe fashion, i.e. axon severing. This is of special interest since previous studies claimed active anterograde transport of Tau protein as one driving force of axonal Tau enrichment.

To validate our system, we assessed the viability and regrowth rates of both neuronal models after axotomy induction. Using a spinning disk confocal microscope setup, we were able to monitor multiple axotomized and control cells in parallel with full coverage on the z-axis. There was no noteworthy neuron loss or detachment for all earlier time points while we observed elevated numbers of dying cells 12 h after axotomy. These results outcompete our previous efforts in SH-SY5Y-derived neurons, which revealed pronounced neuronal loss after axotomy (Bell and Zempel 2022), presumably due to improved attachment and culturing conditions. The grid surface of the cultivation chambers and the modified processing enabled later recognition and immunofluorescent analysis in almost all cases (> 95%).

There are notable differences of the human and mouse neuronal models that have to be considered: 1. The murine neurons are primary neurons. We used cortical neurons and a relatively young embryonal age, E13.5, during development, when only few neuronal cell types/progenitors are reported (for a scRNAseq definition of cortical development see, e.g. Di Bella et al. 2021), but cells are already fated, and we would thus not expect fundamentally different neurons. The specific identity of primary neurons obtained with our culture/differentiation protocol using neuronal media and growth/neurotrophic factors has, to our knowledge, not been established, as this is also an academic question (i.e. how different is the development of neurons in culture, depending on the culture time and condition). In contrast, our human neurons are iPSC-derived, and are differentiated not only with growth factors and neuronal media, but using a genetic switch: Our iPSC-derived neurons are differentiated via forced expression of Ngn2 (inducible via a doxycycline-responsive transactivator), which results in a, compared to other iPSC-based differentiation strategies, very high level of homogeneity of > 90% excitatory glutamatergic cortical neurons (Wang et al. 2017) and may be thus even purer than our already (due to developmental and spatial restriction) quite pure primary murine cortical neurons. 2. The level of maturity is likely very different, with the primary neurons certainly being more mature: While murine cortical neurons are fated at the day of dissection, iPSCs are switched and differentiated by artificially expressing or supplementing neuronal growth factors. Further, murine neurons develop significantly faster (preliminary data suggest approx. 1.5-fold faster AIS maturation) in culture in our hands. 3. We use slightly different media composition for the different cell types, and found that co-cultivation is harmful to the neurons, as can be expected by the high degree of optimization of neuronal cell culture media. As co-cultivation/swapping media will certainly significantly decrease survival, the effect of the co-cultivation is difficult to assess. Other differences in cultivation procedures (e.g. presence of astrocytes only in primary neurons, AraC application only to primary neurons) also applies. 4. Species differences. Tau is a good example, while both species express a rather homologous protein (89% similarity in amino acids), the expression of isoforms and interactors is very different (see e.g. Hernández et al. 2020; Tracy et al. 2022). Thus, while in principle comparable in terms of identity (cortical neurons) and maturity (post-polarity establishment), the two neuronal cell culture systems are not perfectly paired.

Further, we tested whether axon depletion leads to an acute increase of somatic Tau levels or Tau phosphorylation as signs of Tau missorting. Strikingly, neither the somatic Tau levels nor its phosphorylation state were elevated by the acute axon loss in both neuronal model systems. In contrast, we observed a trend (iPSC-derived neurons) and significant effects (mouse primary neurons) towards reduced Tau accumulation and phosphorylation after axotomy. This is surprising as we expected to see accumulation of newly synthesized somatic Tau that cannot transit to the depleted axon. While previous studies postulated the relevance of a retrograde flux of Tau from the axon to the soma, this was refuted by several experimental approaches (e.g. photoconversion, global and specific translation inhibition), all pointing towards newly synthesized Tau as being responsible for AD-like Tau missorting (Zempel et al. 2013, 2017a; Zempel and Mandelkow 2014, 2019) So far, Tau somatic accumulation was considered a consequence of cellular and disease-associated stress, and could e.g. be induced by specific human disease-associated substances (e.g. AD-specific Amyloid-beta oligomers), but also neuron-specific (via excitotoxic stressors such as glutamate) or general cellular stress (elevated calcium, reactive oxygen species) (Zempel et al. 2010), and by dysregulation of the Tau kinase MARK (Chudobová and Zempel 2023), and can be observed in a number of genetic and non-genetic tauopathies (Zimmer-Bensch and Zempel 2021; Al Kabbani et al. 2022). For the exception of very rare genetic forms of tauopathies (like frontotemporal dementia due to mutation in the gene *MAPT*), Tau somatic accumulation is a consequence.

Our findings question the paradigm of protein-based transport as major way for axonal Tau targeting. Indeed, preferential axonal targeting and translation of Tau mRNA was also postulated previously (Litman et al. 1993; Aronov et al. 2001; Morita and Sobuě, 2009). However, several studies suggested only a minor role for anterograde transit of Tau mRNA in neurons (Kosik et al. 1989; Hirokawa et al. 1996; Zempel et al. 2017a; Balaji et al. 2018). An alternative explanation for the absence of Tau accumulation and AT8 phosphorylation in our analysis are compensatory mechanisms evoked by a post-lesion signaling cascade lead to (i) a transcription or translation stop, (ii) acute mRNA degradation, or (iii) selective degradation of Tau and possibly other axon-targeted proteins. The latter process, enhanced somatic degradation of Tau, could also compensate for retrograde diffusion that may derive from the remaining axon stump. Potential leakage of axonal proteins including Tau into the interstitial medium can be excluded since such a phenomenon should have been observed during live-cell monitoring. Possible retrograde Tau missorting could be checked using photoconvertible fluorophores like Dendra2. Multi live-cell monitoring would allow to combine axotomy and compartment specific photoconversion with long-term imaging, but will certainly require careful fine-tuning as current ablation lasers also photoconvert Dendra2 over rather large distances, which might bias or impede accurate quantification of diffused Dendra2.

As mentioned above, the stop or reduction of translation upon axotomy is a plausible scenario. In former studies, Tau mRNA was translocated to stress granules upon stress, which are generally involved in reprogramming the translatome (Moschner et al. 2014), certainly decreasing Tau translation. Conversely, we do not see increases also after short-term (i.e. 0.5–3 h post-axotomy). Hence, remaining Tau mRNA, should still result in continuous Tau production. This means that, while transcriptional regulation may play a role in the long term (< 3 h), we currently cannot confirm this with our setup.

While using a system that is particularly suited for also for long-term imaging (i.e. here used a spinning disc microscope and time-lapse imaging with interval times of 10–20 min), as the total amount of phototoxicity is lower than, e.g. in classical confocal or epifluorescence microscopy, we cannot draw strong conclusions from cells that were analyzed 12 h post-axotomy. We were unable to re-identify most axotomized cells at this time point, indicating that the majority of cells died, resulting in loss of fluorescence marker expression. Of the few cells we could re-identify, however, there was a marked decrease both in total Tau and phospho/AT8-Tau, which could also hint towards a protective downregulation of Tau or Tau-associated kinases. We, however, believe that the accumulation of several stress factors (small number of neurons in imaging chambers), phototoxicity, cultivation in a microscope-located incubator with certainly less stable conditions than a standard incubator, chamber transfer, etc. may be handled well by cells for hours, but, in particular on top of axotomy, not for days. Still, as previously Tau missorting was inducible shortly after stress induction in other paradigms (i.e., as soon as minutes to hours, with the peak usually around 3 h after stress induction Zempel et al. 2010; Zempel et al. 2013; Schützmann et al. 2021; Tjiang and Zempel 2022), one may conclude that simply physically ablating the axon via axotomy is insufficient to induce AD-like Tau missorting as observed previously.

In classical tauopathies like age-related AD or FTD, Tau pathology, which is usually defined by ectopic aggregation and phosphorylation at the AT8 epitope, takes years and decades to develop, which may prompt the assumption that somatic Tau is actually axonal Tau that is redistributed back into the soma (axon-to-soma transit). Experimental evidence, however, recently demonstrated that somatic accumulation of Tau in rodent and human neurons occurs on a timescale of 1–3 h after acute insult with neurotoxic stressors (e.g., Amyloid-beta, mitochondrial impairment), and is not due to axon-to-soma transit, but rather due to the failure of successful routing of newly synthesized Tau into the soma, as shown by photoconversion-pulse-chase and both global and Tau-specific protein synthesis inhibition (Zempel et al. 2010, 2013, 2017a; Tjiang and Zempel 2022). We thus reason that the reaction time of several hours should have been sufficient to result in detectable increase in somatic Tau due impaired routing of newly synthesized Tau into the axon. We hence postulate that axotomy may lead to decreased protein synthesis of Tau.

Besides deciphering Tau sorting mechanisms, our model might have another major benefit for tauopathy-focused research. A small but prominent subset of tauopathies, such as traumatic brain injury (TBI) or chronic traumatic encephalopathy (CTE), is caused by acute or chronic mechanical impact (Gennarelli et al. 1982; Gentleman et al. 1995; Geddes et al. 2000; Tran et al. 2011). In these tauopathies, traumatic axon injury leads to Tau pathology, i.e. hyperphosphorylation, missorting, and aggregation, and ultimately widespread neurodegeneration (Dale et al. 1991; Tokuda et al. 1991; Ikonomovic et al. 2004; Uryu et al. 2007; Goldstein et al. 2012). Our approach bears great potential to complement current model systems as it allows to dissect molecular changes and malfunctions after the axon injury on a single-cell level. Further studies have to target the apparent lack of proper monitoring for several days. This is critical since first signs of Tau pathology after axon injury get visible after several days to even weeks (Ikonomovic et al. 2004; Uryu et al. 2007). However, our system would enable to study rapid effects that precede manifest Tau pathology and that are thought to play a critical role in TBI and CTE, such as misbalance of axonal kinases and phosphatases (Povlishock and Christman 1995; Smith et al. 1999; Tran et al. 2012).

In brief, we developed a laser-based axotomy model in human iPSC-derived and murine primary neurons that allows for investigating effects of acute axon depletion by both multi live-cell monitoring and post-fixation staining. We used this approach to study the impact on different Tau sorting mechanisms, and we could demonstrate the suitability of our system for Tau sorting research in two highly relevant model systems. The results point towards no crucial role of physical axon damage for the development of AD-like somatic Tau accumulation and hyperphosphorylation. However, further experiments are necessary to draw more corroborated conclusions.

## Supplementary Information

Below is the link to the electronic supplementary material.

Supplementary file1 (PDF 278 kb)

## Data Availability

The datasets generated during and/or analyzed during the current study are available in the G-Node repository, https://gin.g-node.org/mbesi94/Axotomy_SomaticTau_Analysis.
