# Supplementary material for: Laser-Induced Axotomy of Human iPSC-Derived and Murine Primary Neurons Decreases Somatic Tau and AT8 Tau Phosphorylation: A Single-Cell Approach to Study Effects of Acute Axonal Damage
Source: Cell Mol Neurobiol. 2023 May 12;43(7):3497–510. doi: 10.1007/s10571-023-01359-z (PMC10477226; doi:10.1007/s10571-023-01359-z)
Supplement: Supplementary file 1 — Supplementary file1 (PDF 278 kb) [file 10571_2023_1359_MOESM1_ESM.pdf]

Laser-induced axotomy of human iPSC-derived and murine primary neurons decreases somatic TAU and AT8 TAU phosphorylation: a single cell approach to study effects of acute axonal damage

Bell-Simons, M.[1,2], Buchholz, S.[1,2], Klimek, J.[1,2], Zempel, H.[1,2]

[1] Institute of Human Genetics, University Hospital Cologne, Kerpener Str. 34, 50931 Cologne, Germany.

[2] Center for Molecular Medicine Cologne (CMMC), Robert-Koch-Str. 21, 50931 Cologne, Germany.

Correspondence:

Hans Zempel, Institute of Human Genetics, University Hospital Cologne, Kerpener Str. 34, 50931 Cologne, Germany, hans.zempel@uk-koeln.de

+++ Supplemental Material +++

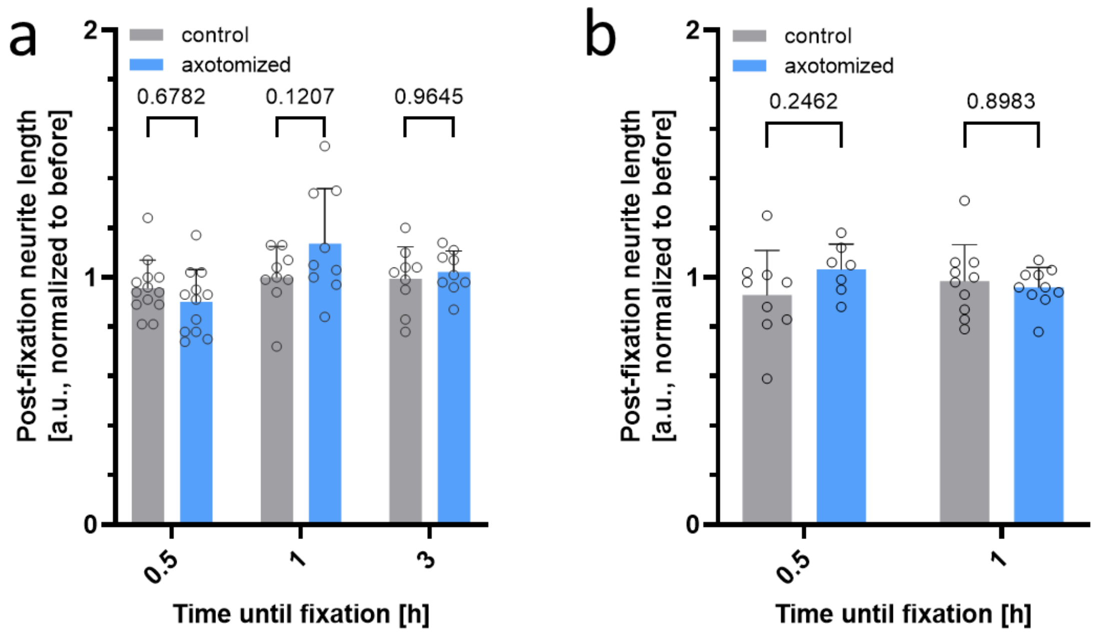

Figure S1: No apparent neurite retraction after axotomy. Human iPSC-derived and mouse primary neurons were fixed after axotomy and subsequent monitoring at different time points (see methods for details). The length of intact neurites of tdTomato-positive axotomized and non-axotomized neurons (a: iPSC-derived neurons, b: mouse primary neurons) was assessed after axotomy, live-cell monitoring, and fixation, and then normalized to the length before the experimental procedure. For each neuron, the values of up to three neurites were averaged. No significant changes are but significant increase of total Tau levels after 1 hour and Tau phosphorylation after 0.5 hours in tdTomato-positive control neurons that is absent in tdTomato-positive axotomized neurons. Grey dots represent individual neurons, colored bars show the arithmetic mean of all neurons, error bars represent the standard deviation (SD). An ordinary two-way ANOVA with Sidak’s multiple comparison test was performed for the determination of significant differences. Exact adjusted p-values are given for all comparisons. For detailed test statistics: see Supplemental Material 1.

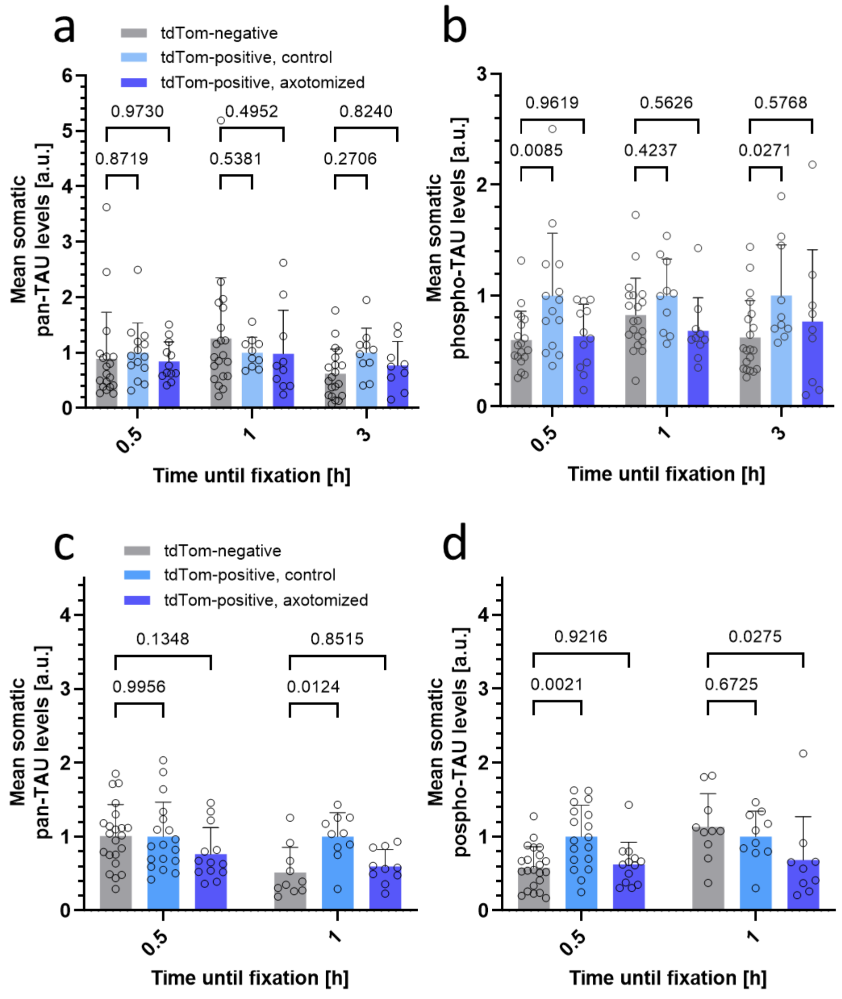

Figure S2: Impact of tdTomato overexpression on somatic Tau accumulation and hyperphosphorylation. Human iPSC-derived and mouse primary neurons were fixed after axotomy and subsequent monitoring at different time points (see methods for details). All cultures were immunostained with a polyclonal anti-panTau antibody (K9JA) and a monoclonal antibody recognizing Tau phosphorylation (AT8 epitope). A & B: Quantification of the total Tau (A) and phospho-Tau (B) signal intensity in the somata of tdTomato-positive axotomized and non-axotomized iPSC-derived neurons, normalized to the signals of tdTomato-negative neurons. Note the modest but significant increase of Tau phosphorylation after 0.5 and 3 hours in tdTomato-positive control neurons that is absent in tdTomato-positive axotomized neurons at both timepoints. C & D: Quantification of the total Tau (C) and phospho-Tau (D) signal intensity in the somata of tdTomato-positive axotomized and non-axotomized primary neurons, normalized to the signals of tdTomato-negative neurons. Note the modest but significant increase of total Tau levels after 1 hour and Tau phosphorylation after 0.5 hours in tdTomato-positive control neurons that is absent in tdTomato-positive axotomized neurons. Grey dots represent individual neurons, colored bars show the arithmetic mean of all neurons, error bars represent the standard deviation (SD). An ordinary two-way ANOVA with Sidak's multiple comparison test was performed for the determination of significant differences. Exact adjusted p-values are given for all comparisons. For detailed test statistics: see Supplemental Material 1.

Supplemental Material 1: Statistical test overview

Fig. 3

c) $F_{interaction}$ (2, 59) = 0.2394, P=0.7879; $F_{row\ factor}$ (2, 59) = 0.2394, P=0.7879;

$F_{column\ factor}$ (1, 59) = 1.190, P=0.2799. Shapiro-Wilk normality test results: control: P (30 min) = 0.0392, P (1 h) = 0.4925, P (3 h) = 0.3651; axotomized: P (30 min) = 0.2709, P (1 h) = 0.0346, P (3 h) = 0.7267

d) $F_{interaction}$ (2, 59) = 0.1172, P=0.8896; $F_{row\ factor}$ (2, 59) = 0.1172, P=0.8896;

$F_{column\ factor}$ (1, 59) = 1.190, P=0.0093. Shapiro-Wilk normality test results: control: P (30 min) = 0.0434, P (1 h) = 0.6016, P (3 h) = 0.0288; axotomized: P (30 min) = 0.2575, P (1 h) = 0.0163, P (3 h) = 0.1577

e) $F_{interaction}$ (2, 59) = 0.8095, P=0.4500; $F_{row\ factor}$ (2, 59) = 0.8109, P=0.4494;

$F_{column\ factor}$ (1, 59) = 1.020, P=0.3167. Shapiro-Wilk normality test results: control: P (30 min) = 0.0026, P (1 h) = 0.5556, P (3 h) = 0.1183; axotomized: P (30 min) = 0.2101, P (1 h) = 0.0055, P (3 h) = 0.4600

f) $F_{interaction}$ (2, 33) = 8.150, P=0.0013; $F_{row\ factor}$ (1, 33) = 11.65, P=0.0017;

$F_{column\ factor}$ (2, 33) = 3.609, P=0.0382. Shapiro-Wilk normality test results: control: P (1 h) = 0.4925, P (3 h) = 0.3651; axotomized + no regrowth: P (1 h) = 0.0691, P (3 h) = 0.5907, axotomized + regrowth: P (1 h) = N too small, P (3 h) = 0.9989

g) $F_{interaction}$ (2, 33) = 2.340, P=0.1121; $F_{row\ factor}$ (1, 33) = 1.109, P=0.3001;

$F_{column\ factor}$ (2, 33) = 1.548, P=0.2278. Shapiro-Wilk normality test results: control: P (1 h) = 0.6016, P (3 h) = 0.0288; axotomized + no regrowth: P (1 h) = 0.7683, P (3 h) = 0.5387, axotomized + regrowth: P (1 h) = N too small, P (3 h) = 0.6119

Fig. 4

c) $F_{interaction}$ (1, 47) = 0.5893, P=0.4465; $F_{row\ factor}$ (1, 47) = 0.5893, P=0.4465;

$F_{column\ factor}$ (1, 47) = 8.623, P=0.0051. Shapiro-Wilk normality test results: control: P (1 h) = 0.0787, P (3 h) = 0.5014; axotomized: P (1 h) = 0.0477, P (3 h) = 0.5795

d) $F_{interaction}$ (1, 46) = 0.05821, P=0.8104; $F_{row\ factor}$ (1, 46) = 0.06044, P=0.8069;

$F_{column\ factor}$ (1, 46) = 7.996, P=0.0069. Shapiro-Wilk normality test results: control: P (1 h) = 0.6084, P (3 h) = 0.6779; axotomized: P (1 h) = 0.0244, P (3 h) = 0.0044

e) $F_{interaction}$ (1, 47) = 1.526, P=0.2229; $F_{row\ factor}$ (1, 47) = 1.526, P=0.2229;

$F_{column\ factor}$ (1, 47) = 0.0008716, P=0.9766. Shapiro-Wilk normality test results: control: P (1 h) = 0.4334, P (3 h) = 0.4786; axotomized: P (1 h) = 0.0450, P (3 h) = 0.0197

f) $F_{interaction}$ (2, 45) = 0.2977, P=0.7439; $F_{row\ factor}$ (1, 45) = 0.8715, P=0.3555;

$F_{column\ factor}$ (2, 45) = 4.210, P=0.0211. Shapiro-Wilk normality test results: control: P (0.5 h) = 0.0787, P (1 h) = 0.5014; axotomized + no regrowth: P (0.5 h) = 0.1136, P (1 h) = 0.5861, axotomized + regrowth: P (0.5 h) = 0.3624, P (1 h) = 0.6923

g) $F_{interaction}$ (2, 44) = 0.4107, P=0.6657; $F_{row\ factor}$ (1, 44) = 0.0001147, P=0.9915;

$F_{column\ factor}$ (2, 44) = 3.844, P=0.0289. Shapiro-Wilk normality test results: control: P (0.5 h) = 0.6116, P (1 h) = 0.6779; axotomized + no regrowth: P (0.5 h) = 0.0656, P (1 h) = 0.0447, axotomized + regrowth: P (0.5 h) = 0.1251, P (1 h) = 0.0448

Fig. S1

a) $F_{interaction}$ (2, 55) = 2.519, P=0.0898; $F_{row\ factor}$ (2, 55) = 5.519, P=0.0065;

$F_{column\ factor}$ (1, 55) = 0.9974, P=0.3223. Shapiro-Wilk normality test results: control: P (0.5 h) = 0.1562, P (1 h) = 0.0841, P (3 h) = 0.7850; axotomized: P (0.5 h) = 0.3954, P (1 h) = 0.4553, P (3 h) = 0.8053;

b) $F_{interaction}$ (1, 32) = 2.051, P=0.1618; $F_{row\ factor}$ (1, 32) = 0.02963, P=0.8644;

$F_{column\ factor}$ (1, 32) = 0.7773, P=0.3845. Shapiro-Wilk normality test results: control: P (1 h) = 0.7176, P (3 h) = 0.4035; axotomized: P (1 h) = 0.9915, P (3 h) = 0.3705;

Fig. S2

a) $F_{interaction}$ (4, 115) = 0.8231, P=0.5130; $F_{row\ factor}$ (2, 115) = 1.564, P=0.2138;

$F_{column\ factor}$ (2, 115) = 0.3209, P=0.7262. Shapiro-Wilk normality test results: positive+control: P (0.5 h) = 0.0392, P (1 h) = 0.4925, P (3 h) = 0.3651; positive+axotomized: P (0.5 h) = 0.2709, P (1 h) = 0.0346, P (3 h) = 0.7267; negative: P (0.5 h) = 0.0001, P (1 h) = 0.0001, P (3 h) = 0.1116;

b) $F_{interaction}$ (4, 115) = 0.6343, P=0.6390; $F_{row\ factor}$ (2, 115) = 0.5490, P=0.5790; $F_{column\ factor}$ (2, 115) = 7.809, P=0.0007. Shapiro-Wilk normality test results: positive+control: P (0.5 h) = 0.0434, P (1 h) = 0.6016, P (3 h) = 0.0288; positive+axotomized: P (0.5 h) = 0.2575, P (1 h) = 0.0163, P (3 h) = 0.1577; negative: P (0.5 h) = 0.1188, P (1 h) = 0.2227, P (3 h) = 0.0152;

c) $F_{interaction}$ (2, 77) = 2.807, P=0.0665; $F_{row\ factor}$ (1, 77) = 6.095, P=0.0158; $F_{column\ factor}$ (2, 77) = 4.537, P=0.0137. Shapiro-Wilk normality test results: positive+control: P (1 h) = 0.0787, P (3 h) = 0.5014; positive+axotomized: P (1 h) = 0.0477, P (3 h) = 0.5795; negative: P (1 h) = 0.5950, P (3 h) = 0.0527;

d) $F_{interaction}$ (2, 76) = 3.992, P=0.0225; $F_{row\ factor}$ (2, 76) = 5.074, P=0.0272; $F_{column\ factor}$ (2, 76) = 4.584, P=0.0132. Shapiro-Wilk normality test results:
positive+control: P (1 h) = 0.6084, P (3 h) = 0.6779;
positive+axotomized: P (1 h) = 0.0244, P (3 h) = 0.0044;
negative: P (1 h) = 0.4409, P (3 h) = 0.5124;
